# Changes of Volatile Flavor Compounds in Sea Buckthorn Juice during Fermentation Based on Gas Chromatography–Ion Mobility Spectrometry

**DOI:** 10.3390/foods11213471

**Published:** 2022-11-01

**Authors:** Dan Wu, Qile Xia, Huan Cheng, Qichun Zhang, Yanbin Wang, Xingqian Ye

**Affiliations:** 1 College of Biosystems Engineering and Food Science, National-Local Joint Engineering Laboratory of Intelligent Food Technology and Equipment, Zhejiang Key Laboratory for Agro-Food Processing, Integrated Research Base of Southern Fruit and Vegetable Preservation Technology, Zhejiang International Scientific and Technological Cooperation Base of Health Food Manufacturing and Quality Control, Fuli Institute of Food Science, Zhejiang University, Hangzhou 310058, China; 2 Food Science Institute, Zhejiang Academy of Agricultural Sciences, Key Laboratory of Post-Harvest Handling of Fruits, Hangzhou 310021, China; 3 Zhejiang Provincial Key Laboratory of Agricultural Resources and Environment, Key Laboratory of Environment Remediation and Ecological Health, Ministry of Education, Zhejiang University, Hangzhou 310058, China; 4 Zhejiang Academy of Forestry, Hangzhou 310023, China

**Keywords:** *Lactobacillus*, *Saccharomyces cerevisiae*, GC–IMS, sensory evaluation, fingerprint map

## Abstract

Sea buckthorn is rich in polyphenolic compounds with antioxidant activities. However, it is very sour, and its odor is slightly unpleasant, so it requires flavor improvement. Fermentation is one potential method. Sea buckthorn juice was fermented at 37 °C for 72 h and then post-fermented at 4 °C for 10 days. The flavor-related properties of the sea buckthorn juice were evaluated during fermentation, including the pH, total soluble solids (TSS), color, sensory evaluation, and volatile flavors. The sea buckthorn fermented juice had a low pH. The total soluble solids decreased from 10.60 ± 0.10% to 5.60 ± 0.12%. The total color change was not more than 20%. Fermentation increased the sweet odor of the sea buckthorn juice, but the fruity flavor decreased and the bitter flavor increased. A total of 33 volatile flavors were identified by headspace gas chromatography–ion mobility spectrometry (GC–IMS), including 24 esters, 4 alcohols, 4 terpenes, and 1 ketone. Their total relative contents were 79.63–81.67%, 10.04–11.76%, 1.56–1.22%, and 0.25–0.55%, respectively. The differences in the characteristic volatile molecular species of the sea buckthorn juice at different fermentation stages could be visually discerned using fingerprint maps. Through principal component analysis (PCA), the total flavor difference of the sea buckthorn juice at different fermentation stages could be effectively distinguished into three groups: the samples fermented for 0 h and 12 h were in one group, the samples fermented for 36 h, 48 h, 60 h, and 72 h were in another group, and the samples fermented for 24 h were in another group. It is suggested that sea buckthorn juice be fermented for 36 h to improve its flavor. GC–IMS and PCA are effective methods of identifying and distinguishing the flavor characteristics of sea buckthorn juice. The above results can provide a theoretical basis for studying the changes in sea buckthorn’s characteristics as a result of fermentation, particularly with regard to its flavor.

## 1. Introduction

Sea buckthorn (*Hippophae rhamnoides* L.) is a deciduous shrub that belongs to the Elaeagnaceae family. It adapts to poor soils, is tolerant to cold, heat, and drought, and can grow in deserts [1,2]. Many studies over the past decade have shown that sea buckthorn berries are beneficial to people’s health. They are rich in phenolic compounds, polysaccharides, vitamins, fatty acids, and other natural nutrients, that may have antioxidant, antibacterial, and antiviral properties; they may also have a positive effect on some chronic diseases [3,4,5,6,7,8]. China and Mongolia are the leading producers, and China has the largest area of sea buckthorn cultivation [1,9]. There are six species and 12 subspecies of sea buckthorn, among which five species and eight subspecies are distributed in China [10].

Fermentation is considered to be a processing method that helps to maintain and improve the nutrient and sensory properties of food. Fermentation can produce some functional substances during metabolism, which is beneficial to improve the quality of fruit juice and produces a strong aroma [11,12]. It has been reported that fermentation can significantly improve the antioxidant capacity, as well as the phenols and aroma components, of fruit juices [13,14,15,16,17]. Schubertová et al. reviewed the effect of malolactic fermentation on the organic acids and polyphenols of sea buckthorn fruit juice. Malolactic fermentation improved its sensory attributes and enhanced its antioxidant activity [5,18,19]. When adding sea buckthorn material to soy milk, probiotic (*Lactobacillus casei* subsp. *Paracasei*) viability is enhanced and the sensory qualities of soy milk are improved [20]. A co-fermented product of sea buckthorn with *S. cerevisiae* and *I. orientalis* has been studied. The malic acid degraded with fermentation, and the total phenolic content of the co-fermented product was higher in comparison to commercial red wines. It also exhibited significant anti-oxidation activity. Sea buckthorn is rich in polyphenolic compounds with antioxidant activities [21], but it is very sour, and the odor is slightly unpleasant, so its flavor requires improvement. Fermentation may be a potential method to improve its sensory qualities, including its flavor. *Lactobacillus* fermentation mainly produces acids and aldehydes, whereas *S. cerevisiae* mainly produces alcohols and ketones [22,23,24,25]. *Lactobacillus* can degrade alcohol [26]. The competition with yeast cells for essential growth factors was a reason for decreases in final ethanol yield [27]. A prion, [GAR+], that let yeast cells make less ethanol and bypass quintessential yeast metabolism, allowed cells to utilize other carbon sources as a carbon source even in the presence of glucose [28]. Some lactic acid bacteria were able to induce the [GAR+] prion that led into less alcohol production [29]. Chan et al. found that the mixed culture of *L. paracasei* and *S. cerevisiae* possessed the volatile ethyl lactate, but it was not found in the single cultures of either *L. paracasei* or *S. cerevisiae* [30]. The presence of lactic acid bacteria may convert some alcohols into ester functions. Wu et al. found that *L. paracasei* was able to stimulate malolactic fermentation. Compared with other strains, the relative content of alcohols obtained by fermentation with this strain was the lowest [31]. At the same time, studies on the *L. paracasei* fermentation of sea buckthorn juice is rare, so it is necessary to explore its effect on fermentation. Light fermentation with *L. paracasei* and *S. cerevisiae* may inhibit the growth rate of alcohols and improve the flavor. Sea buckthorn juice has low pH value and low sugar content [32,33], so adding proper amount of sucrose can promote fermentation.

The methods of detecting flavor compounds can be divided into sensory analysis and instrument analysis. Instrument analysis methods include gas chromatography–mass spectrometry (GC–MS), gas chromatography–olfactometry (GC–O), liquid chromatography–mass spectrometry (LC–MS), gas chromatography–ion mobility spectrometry (GC–IMS), and so on [34,35,36,37]. GC–MS has become a common method for the detection of volatile compounds in food flavor due to its advantages of high sensitivity, low detection limit, and accurate qualitative analysis [38,39,40]. GC–IMS is a rapid detection technique for volatile organic compounds that combines the advantages of the high separation performance of gas chromatography with the fast response and high sensitivity of the ion migration spectrum [41]. Compared with GC–MS, GC–IMS has the advantage of simple operation and no vacuumization. It can detect volatile components and separate the isomers of volatile components under atmospheric pressure [42]. At present, there are few reports on the aroma and flavor analysis of sea buckthorn using ion migration spectrometry. In this study, changes in the flavor compounds in the sea buckthorn juice fermentation process were studied using GC–IMS. The fingerprint of the volatile odor of sea buckthorn juice at different fermentation stages was established, and the volatile flavor components of sea buckthorn juice were visualized, which provided information about the flavor change rules of sea buckthorn juice during fermentation.

## 2. Materials and Methods

### 2.1. Materials

Frozen sea buckthorn (named “shengqiuhong”, *Fructus hippophae*, produced in Tacheng, Xinjiang, China) was provided by Gansu Aikang Sea buckthorn Products Co., Ltd. (Dingxi, China). *L. paracasei* was provided by Xi’an Jushengyuan Biotechnology Co., Ltd. (Xi’an, China). *S. cerevisiae* was provided by Angelyeast Inc. (Yichang, China). Sucrose was from the local market.

### 2.2. Fermentation Treatment

500 g sea buckthorn was cleaned with sterile water, taken into the Homogenizer (DS-1, Shanghai Specimen and Model Factory, China) to make juice, and then put into the fermentation tanks (sterilization). 500 g of sterile water, 50 g of sugar, 0.75 g of *S. cerevisiae*, 2.5 g of *L. paracasi* were added into the sea buckthorn juice and mixed uniformity. Under anaerobic conditions, the sea buckthorn juice fermented for 0 h, 12 h, 24 h, 36 h, 48 h, 60 h, and 72 h at 37 °C in an incubator at first, and then they were stored 10 days at 4 °C in a refrigerator for post fermentation. After that, the samples were filtered by 400 mesh filter cloth and put into centrifuge tube and stored in −20 °C refrigerator. The pH, total soluble solids (TSS), color, sensory evaluation and volatile flavors were evaluated. Three replicates have been performed.

### 2.3. pH and TSS

The pH of the sea buckthorn fermented juice was quantified by a Mettler automatic titrator (FE-28, Mettler-Toledo Instruments Co., Ltd., Shanghai, China). TSS of the sea buckthorn fermented juice was measured by a calibrated digital refractometer (LH-T55, Zhejiang Lohand Environmental Technology Co. Ltd., Hangzhou, China).

### 2.4. Color

The color of the sea buckthorn fermented juice was evaluated by the CIELab chroma system [43]. The color measurement was carried out by a high-quality Colorimeter (SC-10, 3NH Technology Co., Ltd., Guangdong, China). The CIE-L a b scale was used to evaluate the color in the study, where L value meant brightness, a value meant redness, b value meant yellowness. The total color chromaticity (E) was obtained by the following equation:

(1) $$E=\sqrt{a^{2}+b^{2}+L^{2}}$$

### 2.5. Sensory Evaluation

Quantitative descriptive analysis was carried out according to the reported method at 25 ± 1 °C sensory panel room [44]. The sensory properties of sea buckthorn fermented juice were evaluated by a panel of 12 assessors, six males and six females, aged from 20 to 45 years. The panelists had been trained according to national standards s ISO 8586 and ISO 6658 prior to the sensory evaluation. A 9-point linear intensity scale ranging from 0 to 9 was used (Supplementary Table S1). Approximately 10 mL of juice was served into odor-free, disposable, transparent 30 mL plastic cups to each panelist, along with the questionnaire, one at a time, with about 15 min wait between samples. Each sample was assessed in triplicate, and the mean of each sample was expressed by the average of the three scores based on a 9-point scale. The statistical analysis of *t*-test was used to compare the recorded data of repeated panel performances to validate the reliability of the intensity scale.

### 2.6. Identifification of Flavor Compounds by GC–IMS

The sea buckthorn fermented juice was identified for flavor compounds by GC–IMS, as described by Lin et al. [45] with slight modifications.

The GC–IMS analysis was carried out by a FlavourSpec^®^ GC–IMS system (G.A.S Company, Berlin, Germany). 1 mL of the sea buckthorn fermented juice was placed into a 20 mL headspace vial and then incubated at 40 °C for 30 min at 500 rpm before injecting. The injection volume was 500 µL, and the injection temperature was 85 °C. The separation of the compounds was carried out on Restek MXT-5 column (0.53 mm × 15 m, 1 µm; Restek Corporation, Bellefonte, PA, USA). Column temperature was maintained at 60 °C. Nitrogen gas (>99.999%) was used, gradient profile: 0–2 min, 2 mL/min; 2–10 min, 2–10 mL/min; 10–20 min, 10–100 mL/min; 20–30 min, 100–150 mL/min; 30–35 min, 150 mL/min. IMS module: the temperature of IMS detector was 45 °C. The carrier gas was nitrogen (≥99.999%), and the flow rate of carrier gas was 150 mL/min.

The retention index (RI) of each compound was calculated using n-ketones C4–C9 (Sinopharm Chemical Reagent Beijing Co., Ltd., Beijing, China) as external references and the calculations were performed by the automated mass spectral deconvolution and identification system. The volatile flavor compound was identified by comparing the RIs and drift times. Each spectrum was reported as an average of 13 scans. The formula of the relative content of each volatile flavor compound was specified as:

relative content (%) = Ax/At

where “Ax” meant the peak areas of each volatile flavor compound in a sample, “At” meant the sum of peak areas of all volatile compounds in a sample.

### 2.7. Statistical Analysis

All measurements were carried out three times in parallel; the results were expressed as the mean ± standard deviation. Data of GC–IMS was performed with the GC–IMS instrument’s analysis software, which included a laboratory analytical viewer and three plug-ins (Reporter, Gallery plot, Dynamic principal component analysis). Statistical analysis (ANOVA and Duncan multiple range test) was performed with the Data Processing System (DPS) software v18.10 [46].

## 3. Results and Discussion

### 3.1. pH and TSS Changes in Sea Buckthorn Juice during Fermentation

**Figure 1:**
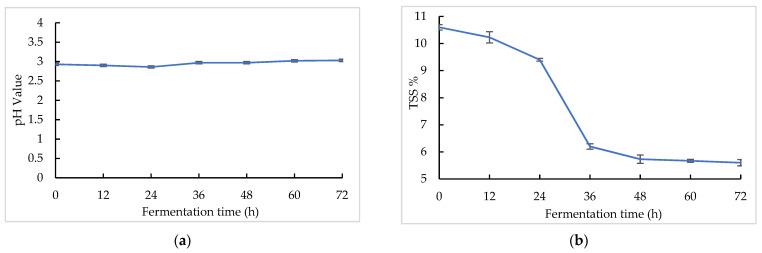
pH and TSS changes in sea buckthorn juice during fermentation: (**a**) pH change; (**b**) TSS change.

The pH and TSS changes in the sea buckthorn juice during fermentation are shown in Figure 1. The sea buckthorn juice had a low pH [47]. The pH values decreased in the first 24 h from 2.90 ± 0.01 to 2.86 ± 0.01, and after that it increased gradually from 2.86 ± 0.01 to 3.03 ± 0.01 with fermentation. At the same time, the TSS values decreased over the whole fermentation period from 10.60 ± 0.10% to 5.60 ± 0.12%. The majority of the TSS decline occurred between 24 and 36 h. The microorganism may have entered the logarithmic growth stage at this time. *Lactobacillus* is a malolactic organism. Malolactic fermentation could convert malic acid into lactic acid, which leads to a higher pH and lower acidity. Wu et al. found that six lactic acid bacteria, including *L. paracasei*, exhibited a strong capacity to convert malic acid to lactic acid [31]. Fu et al. found that the contents of malic acid and citric acid in sea buckthorn juice decreased with lactic acid bacteria fermentation, while the content of lactic acid increased, so lactic acid bacteria had the capacity to metabolize citric acid [48]. The change in the pH value of sea buckthorn fermented juice may have been caused by the organic acid metabolism of microorganisms. Both *S. cerevisiae* and *Lactobacillus* consume a lot of sugar during fermentation, which may have caused the decline in the TSS [26].

### 3.2. Color Changes in Sea Buckthorn Juice during Fermentation

**Figure 2:**
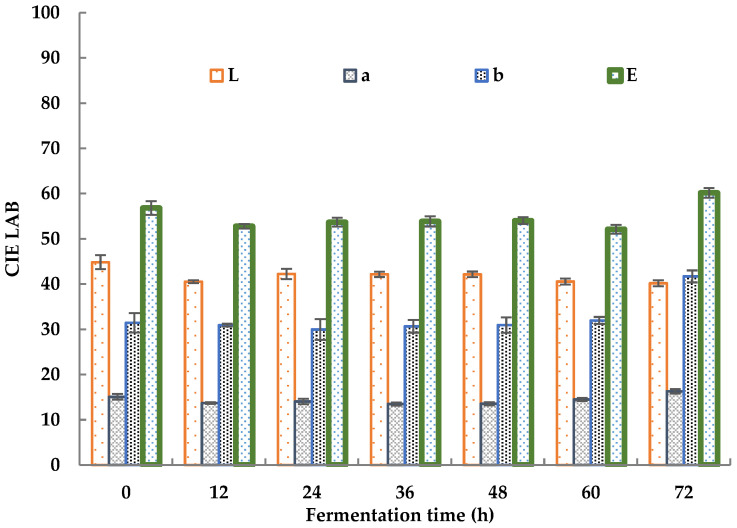
Color changes in sea buckthorn juice during fermentation.

The color changes in sea buckthorn juice during fermentation is shown in Figure 2.

The total color chromaticity (E) decreased from 56.82 ± 2.33 to 52.78 ± 0.49 in the first 12 h of fermentation. It slightly increased to 53.85 ± 0.95 after 24 h of fermentation and then held a steady value from 24 h to 48 h, decreased to 52.13 ± 0.98 after 60 h, and increased to 60.16 ± 1.16 after 72 h. The decrease in E during the initial 12 h was mainly caused by a decrease in the red value (a) and light value (L). The red value showed a decrease from 15.07 ± 0.63 to 13.70 ± 0.19, and the light value decreased from 44.84 ± 1.53 to 40.52 ± 0.32. The proliferation of microorganisms led to an increase in the turbidity of the sea buckthorn juice, which led to a decrease in the L value. The consumption of nutrients by microorganisms led to a decrease in the turbidity of the sea buckthorn juice, which led to an increase in the L value [49]. The changes in the a and b values were due to changes in some colored compounds, including polyphenols, flavonoids, carotene, and so on [47]. The increase in E after 72 h was mainly caused by an increase in the blue value (b), which increased from 31.96 ± 0.77 to 41.69 ± 1.33. He et al. revealed the chromogenic mechanism of sea buckthorn. Its fruit color depended mainly on the ratio of lycopene to β-carotene, and the content of carotene was more than eight times that of lycopene [50]. Changes in the L, a, and b value showed the same trend as carrot powder before and after fermentation as reported by Ma, which was rich in β-carotene [51]. Taking the sample fermented for 0 h as a reference, the color differences between the samples fermented for 24 and 48 h and the reference sample were smaller than 5.53%. Thus, a fermentation period of 24–48 h is recommended for sea buckthorn juice on the basis of its color.

### 3.3. Sensory Changes in the Sea Buckthorn Juice during Fermentation

**Figure 3:**
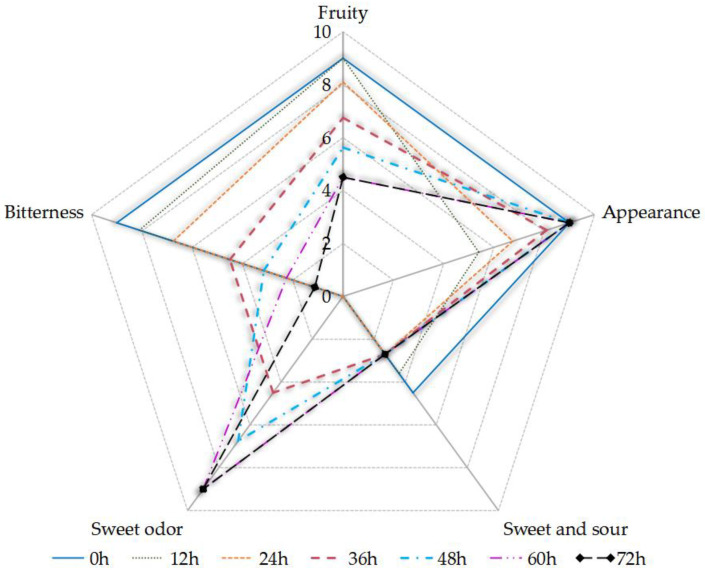
The sensory evaluation of the sea buckthorn juice during fermentation.

The sensory assessors reported that the fruity flavor and sweet–sour taste of the sea buckthorn juice decreased, that the sweet odor became stronger, and that the bitterness increased with fermentation time (Figure 3). The full score of each sensory index was 9 points. Fermentation did not promote the sensory qualities of the fermented juice except the sweet odor. The sensory intensity of “sweet and sour” decreased due to the microbial consumption of TSS, which was mainly caused by the sucrose content and the small change in pH during fermentation (Figure 1). The phenolic acids and flavonoids in the sea buckthorn juice might be the main reason for its bitterness, including gallic acid [36,52]. An ethyl, β-d-glucopyranoside, that was present in the sea buckthorn, contributed to its bitterness [53]. The sensory intensity of “sweet odor” increased due to the esters and alcohols in the samples, including ethanol and ethyl octanoate (see Table S1). *S. cerevisiae* was an important producer of ethanol and ethyl octanoate. Alcohols were produced from glucose degradation and amino acid catabolism, causing a sweet odor. The ethyl octanoate provided a winey, sweet, apricot, banana, brandy, pear odor, which were important components of the aroma of the fermented juice [26,54]. The malolactic fermentation of *Lactobacillus* can convert malic acid into lactic acid, which leads to a higher pH and lower acidity. The lactic acid had a milder taste than the malic acid. *Lactobacillus* is involved in the metabolism of flavor substances in sea buckthorn juice fermentation, which can improve the flavor of the sea buckthorn juice [49].

### 3.4. Volatile Substances Changes in the Sea Buckthorn Juice during Fermentation

#### 3.4.1. GC–IMS Analysis for Characteristic Peak of Volatile Flavor Compounds during Sea Buckthorn Juice Fermentation

**Figure 4:**
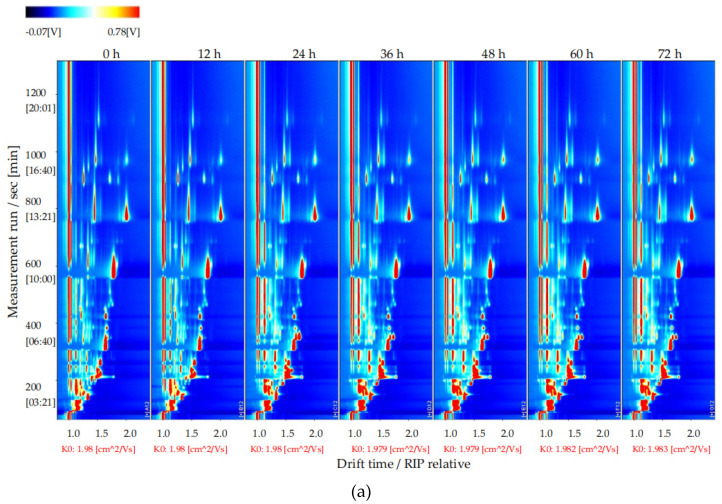

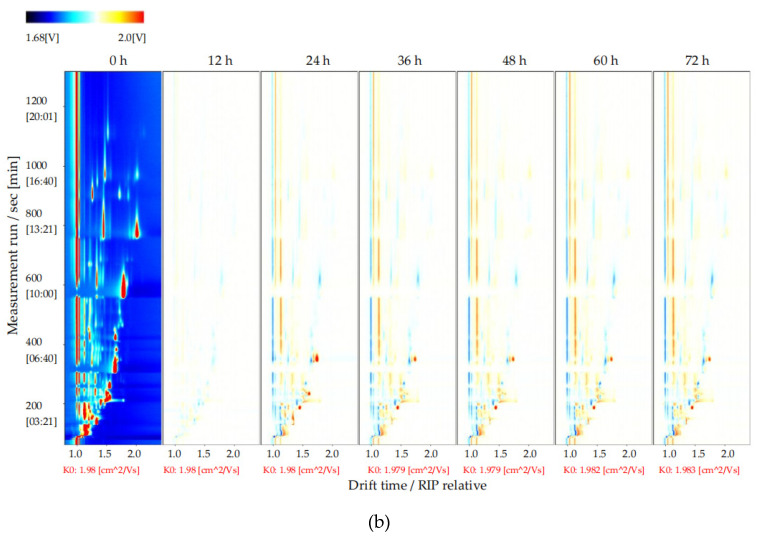
Results of GC−IMS two dimensional spectra for the volatile flavor compounds of the sea buckthorn juice fermented for 0 h, 12 h, 24 h, 36 h, 48 h, 60 h, 72 h. (**a**) Intuitive comparison; (**b**) differences comparison. the *y*-axis was the retention time of GC, the *x*-axis was the drift time of IMS. The red vertical line at 1.0 on the abscissa indicated the reaction ion peak (RIP). The spot on the right side of the RIP was characteristic peak signal of the flavor substances, which represented different volatile flavor substances in the sample.

To explore changes in the volatile flavor compounds in sea buckthorn juice during fermentation, the two-dimensional spectra plot of the GC–IMS results was analyzed (Figure 4). The *y*-axis is the retention time of GC, and the *x*-axis is the drift time of IMS. The red vertical line at 1.0 on the abscissa indicates the reaction ion peak (RIP). The spot on the right side of the RIP is a characteristic peak signal of the flavor substances, which represents different volatile flavor substances in the sample. The color of the spot represents the concentration of the volatile flavor substance; the redder the higher, the whiter the lower. The drift time of all volatile flavor substances was in the range of 1.0–2.0 ms, whereas the retention time was in the range of 90–1150 s.

It is difficult to directly judge the differences in the volatile flavor compounds in the sea buckthorn juice samples with fermentation time from Figure 4a. In order to compare this difference more obviously, the spectral plot of the sea buckthorn juice fermented at 0 h was taken as the reference. The plots of other samples after fermentation were obtained by subtracting the reference plot (Figure 4b). If the concentration of the volatile flavor compound in the sample was the same as the reference, the characteristic peak signal in the plot was white after deduction; if the concentration of the volatile flavor compound was lower than the reference, the characteristic peak signal was blue; if the concentration of the volatile flavor compound in sample was higher than the reference, the characteristic peak signal was red. We can see that the components of the samples of sea buckthorn juice fermented for 12 h and 0 h were basically the same. After 24 h of fermentation, the volatile flavor components in the sea buckthorn juice changed. It can be seen from Figure 4b that some components increased with fermentation, some decreased with fermentation, and some appeared during the fermentation process and then gradually decreased or disappeared.

#### 3.4.2. Identification of Volatile Flavor Compounds in the Sea Buckthorn Fermented Juice

A total of 47 signals were detected in the sea buckthorn juice during fermentation. According to the RI and Dt of the detected signal, 33 signals were identified when compared to the standard reference compounds in the NIST database and the IMS database, which are shown in Table 1. They include 24 esters, 4 alcohols, 4 terpenes and 1 ketone. Monomer, dimer, or polymer were found in some compounds, for example, ethyl hexanoate monomers and dimers, isopentyl alcohol monomers and dimers, and beta-ocimene monomers and polymers. A total of 14 volatile organic compounds were not identified, which had RI values between 675.00 and 1171.90. Ethyl hexanoate (threshold: 5.00 × 10^−4^ mg/kg), ethyl 2-methylbutanoate (threshold: 1.50 × 10^−4^ mg/kg), isopentyl acetate (threshold: 0.003 mg/kg), 6-methyl-5-hepten-2-one (threshold: 0.1 mg/kg), and ethyl 2-methylpropanoate (threshold: 1.00 × 10^−4^ mg/kg) contribute to the aroma of sea buckthorn [2]. These compounds were all detected here and may have contributed to the aroma of the sea buckthorn juice during fermentation. Ethyl hexanoate and ethyl 2-methylbutanoate have low thresholds and a fruity aroma. After fermentation, the amounts of these two substances decreased, which might be the reason for the decline in the fruity odor. In addition to the flavors mentioned above, according to our sensory evaluation and the relative content of volatile compounds in Table 1, the ethyl octanoate (threshold: 1.00 × 10^−4^ mg/kg) and the ethanol (threshold: 2900 mg/kg) might be contribute to the sweet odor. After fermentation, the flavors of the sea buckthorn juice were mainly esters and alcohols, which is consistent with the relevant literature [14,48]. In contrast to the GC–MS method (solid phase microextraction–gas chromatography–mass spectrometry), (E)-2-Hexenyl acetate and propyl acetate were detected via GC–IMS [2,14,48].

**Figure 5:**
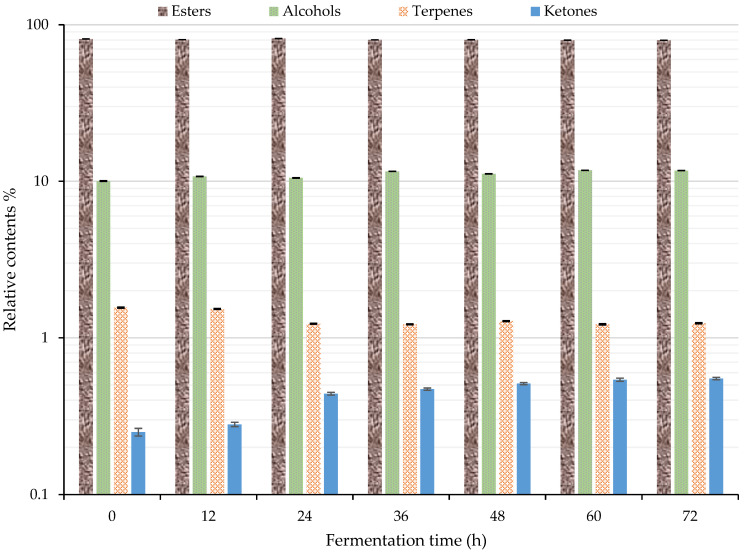
Changes of volatile flavor compounds during the fermentation of sea buckthorn juice by GC–IMS.

The change in the relative content of each category of volatile flavor compound is shown in Figure 5. When combined with Table 1, the total volatile flavor substances identified by GC–IMS accounted for more than 93% of the total. The esters accounted for 79.63–81.67%, followed by alcohols with 10.04–11.76%. The terpenes and ketones accounted for significantly less. Their values were about 1.56–1.22% and 0.25–0.55%, respectively. Furthermore, the 10 most common substances in the esters, from high to low content, were ethyl hexanoate (D, 19.39–23.17%), ethyl 2-methylbutyrate (14.23–15.78%), isopentyl isovalerate (D, 6.98–7.89%), ethyl acetate (5.98–9.43%), ethyl butyrate (4.26–5.73%), isopentyl isovalerate (M, 3.73–5.13%), ethyl hexanoate (M, 2.02–3.35%), ethyl octanoate (M, 2.76–3.10%), ethyl 2-methylpropanoate (1.89–2.86%), and ethyl benzoate (M, 2.09–2.67%). Their total amount accounted for 82.72–92.26% of the relative content of esters. Ethanol had the highest relative content in alcohols, accounting for 73.49–76.63% of alcohol content. Among the esters, ethyl hexanoate, ethyl 2-methylbutanoate, and ethyl octanoate have a fruity aroma. Ethyl hexanoate and ethyl octanoate have a delicate bouquet [2,48].

The contents of ethyl hexanoate and ethyl 2-methylbutanoate in the fermented sea buckthorn juice were larger greater than that of other compounds, and they might be the main contributors to the flavor of the juice. Figure 5 suggests that 24 h might be the key fermentation period. The relative content of esters reached the highest (81.67%) at 24 h of fermentation. The content of terpenes decreased from 1.56% to 1.23% after 24 h of fermentation. After that, the change was not obvious. The content of ketone increased by 76% from 0.25% to 0.44% after 24 h of fermentation. After that, the rate of increase slowed down. The content of alcohols increased from 10.04% to 11.57% between 24 and 36 h of fermentation. After that, the change was not obvious. Lactic acid bacteria can degrade alcohols [26], so the increase in alcohols was due to the *S. cerevisiae*. *Lactobacillus* fermentation mainly produces acids and aldehydes, whereas *S. cerevisiae* mainly produces alcohols and ketones, so the increase in alcohols and ketones was due to *S. cerevisiae*. Isopentyl alcohol is a unique product of *S. cerevisiae* fermentation [22,23,24,25]. The esters produced by *S. cerevisiae* fermentation are more abundant than those produced by *Lactobacillus* fermentation because *S. cerevisiae* fermentation produces more alcohols than *Lactobacillus,* and alcohols continuously esterify to produce esters through fermentation and aging [25]. The material changes during fermentation depend on the growth balance between *S. cerevisiae* and *L. paracasei*.

**Figure 6:**
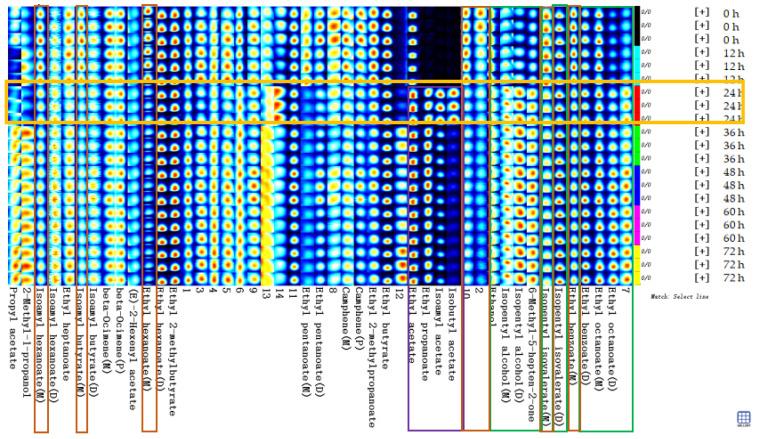
Fingerprints of volatile flavor compounds in the sea buckthorn juice during fermentation. Each line in the graph represented the signal peaks in a sample. Each column represented the signal peak of the same volatile substances measured in triplicate for each storage time, and the unidentified substances in samples were described by numbers.

Figure 6 shows the fingerprints of volatile substances in the sea buckthorn juice during fermentation. Each row in the graph represents all the volatile organic substances in a sample. Each column represents the content difference of the same volatile substance measured in triplicate for each storage time, and the unidentified substances in the samples are labelled with numbers. Black, dark blue, light blue, white, yellow, and red represent the concentration of the substance from low to high, with pure black representing the concentration of a substance close to zero.

Figure 6 describes the complete volatile substances change information for each sample and the differences between samples. The contents of the substances in the green box in Figure 6 increased with fermentation time, including ethyl octanoate (M, from 2.76% to 3.09%; D, from 0.81% to 1.83%), ethyl benzoate (D, from 1.01% to 1.19%), isopentyl isovalerate (D, from 6.98% to 7.89%), 6-methyl-heptene-2-one (from 0.25% to 0.55%), isopentyl alcohol (M, from 2.04% to 2.16%; D, from 0.23% to 0.60%) and ethanol (from 7.59% to 8.62%). The contents of the substances in the red box in Figure 6 decreased with fermentation time, including ethyl 2-methylpropanoate (from 2.70% to 1.92%), ethyl benzoate (M, from 2.66% to 2.18%), isopentyl hexanoate (M, from 1.04% to 0.87%), ethyl pentanoate (D, from 0.98% to 0.68%), and ethyl pentanoate (M, from 0.36% to 0.13%). The contents of the substances in the purple box in Figure 6 increased significantly at a certain stage of fermentation, such as isobutyl acetate, isopentyl acetate, ethyl propanoate, and ethyl acetate. They appeared or increased significantly in the samples after 24 h of fermentation. The formation of the sea buckthorn flavor compounds was related to amino acid metabolism, fatty acid metabolism, malolactic fermentation, β-carotene degradation, and so on, among which some intermediates are produced [2,14,55].

#### 3.4.3. Principal Component Analysis (PCA) of the Characteristic Flavor Compounds Present in the Sea Buckthorn Juice during Fermentation

**Figure 7:**
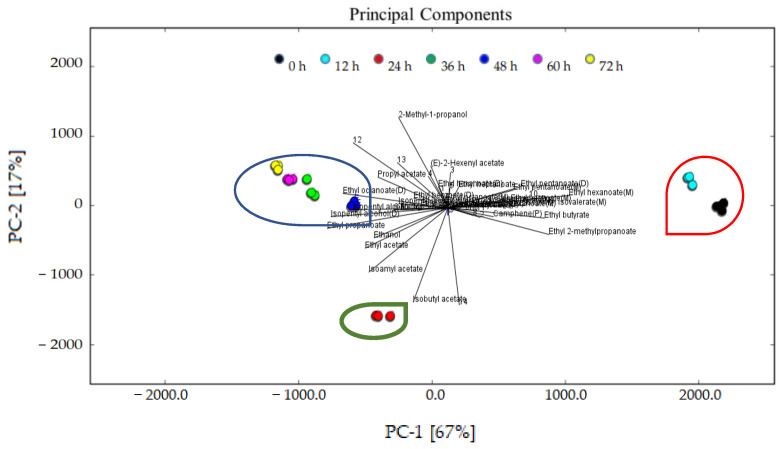
PCA analysis of the characteristic flavor compounds presented in the sea buckthorn juice during fermentation.

A PCA based on the fingerprint map and GC–IMS signal peak spectrum of the volatile organic compounds is shown in Figure 7. It expresses the difference in the flavor characteristics of the sea buckthorn juice at different fermentation stages more directly. In Figure 7, the contribution rate of PC1 was 67%, while the contribution rate of PC2 was 17%. The cumulative variance contribution rate was 84%. The results show that PC1 and PC2 contained a large amount of information about the samples and could reflect the characteristic flavor compounds presented in the sea buckthorn juice during fermentation. The distance between the samples represents the difference; the longer the distance, the greater the difference; the shorter the distance, the smaller the difference. It can be seen that the flavor compounds of the sea buckthorn juice at the fermentation stages of 0 h and 12 h are relatively close, and at the same time the flavor compounds of the sea buckthorn juice at the fermentation stages of 36 h, 48 h, 60 h, and 72 h were also close. The flavor compounds of the sea buckthorn juice fermented for 24 h were quite different from those mentioned above.

The analysis results of the PCA are consistent with those of Figure 5 and Figure 6. The fermentation stage of 24 h was important. With 24 h fermentation, the relative content of esters reached its highest, the rate of decrease of terpenes reached its highest, and the rate of increase of ketone reached its highest. According to Figure 6, some flavor substances increased, including ethyl octanoate, ethyl benzoate (D), isopentyl isovalerate (D), 6-methyl-heptene-2-one, isopentyl alcohol, ethanol, isobutyl acetate, isopentyl acetate, ethyl propanoate, ethyl acetate, and unknown volatiles 7 and 14; in contrast, some flavor substances decreased, including ethyl benzoate (M), isopentyl isovalerate (M), ethyl 2-methylpropanoate, camphene, ethyl pentanoate, ethyl hexanoate (M), isopentyl hexanoate, ethyl heptanoate, and unknown volatiles 10, 1, 3, 4, and 5. This indicates that the microorganisms may have entered the logarithmic growth stage at 24 h of fermentation. After fermentation for 36 h, all kinds of flavor compound substances were relatively stable. We thus recommend 36 h of fermentation to improve sea buckthorn juice flavor. Compared with Figure 3, the sensory profile continues to evolve and has different performance in the fermentation process. The different generation mechanisms of volatile flavors and taste compounds may be the reason.

## 4. Conclusions

Sea buckthorn juice was fermented for 72 h at 37 °C and post-fermented at 4 °C for 10 days. The changes in the characteristic of the sea buckthorn juice during fermentation were studied by physicochemical analysis, sensory evaluation, and gas chromatography–ion mobility spectrometry.

The pH of the sea buckthorn juice during fermentation decreased in the first 24 h from 2.90 ± 0.01 to 2.86 ± 0.01, and after that, it increased gradually from 2.86 ± 0.01 to 3.03 ± 0.01. At the same time, the TSS values decreased over the whole fermentation process from 10.60 ± 0.10% to 5.60 ± 0.12%. The color change was not significant during fermentation. The initial total color chromaticity was 56.82 ± 2.33. At the stage of fermentation between 12 h and 60 h, it maintained a value of 53.29 ± 0.81, and after that, it increased to 60.16 ± 1.16 at 72 h. The proliferation of microorganisms and the microorganism consumption of nutrients were the reasons for the changes in the pH, TSS, and color of the juice [26,31,47,48,49,50].

Sensory evaluation showed that the fruity flavor of the sea buckthorn juice decreased, the sweet odor became stronger, and the bitterness increased with fermentation time. The ethyl hexanoate and the ethyl 2-methylbutanoate had low thresholds, and the decreased content of these two substances might be the reason for the decline in the fruity odor. The increase in ethyl octanoate and ethanol levels might be the reason for the increase in the sweet odor. The phenolic acids, flavonoids, and β-d-glucopyranoside may have contributed to the bitterness [36,52,53].

A total of 47 signals were detected in the sea buckthorn juice during fermentation by GC–IMS. Of these, 33 signals were identified and 14 were not identified. The identified volatiles include 24 esters, 4 alcohols, 4 terpenes and 1 ketone. Their relative content from large to small was esters > alcohols > terpenes > ketone. IMS’ application was not long, and its database is not large enough, so some compounds could not be identified, such as butyl caproate, ethyl 3-methylbutanoate, 3-methylbutyl ester, isobutyl isobutyrate, 3-methyl-butanoic acid, 3-hydroxy-2-butanone, 2-methyl-1-propanol, 3-methyl-1-propanol, 2-methyl-2,4-pentanediol, and benzyl alcohol. These have all been reported in sea buckthorn [2] but are not in the IMS database and were thus not identified here. In future work, gradually enriching the database and improving the detection sensitivity will be the main development direction of IMS.

Fingerprints were developed that allowed us to visually discern the differences in the characteristic volatile molecular species of the sea buckthorn juice at different fermentation stages. Through principal component analysis, the total flavor difference of the sea buckthorn juice at different fermentation stages could be effectively distinguished. The results show that the stage of fermentation at 24 h was important. Microorganisms may have entered the logarithmic growth stage at this time. With 24 h fermentation, the relative content of esters reached its highest, the rate of decrease of terpenes reached its highest, and the rate of increase of ketones reached its highest. Some flavor substance increased but some flavor substances decreased too. *S. cerevisiae* and *Lactobacillus* were important producers of flavor and odor. Based on the PCA analysis, we suggest that fermentation take place for 36 h to improve the flavor of sea buckthorn juice.

As a method of flavor detection, IMS can detect flavor components in the absence of sample pretreatment. It is sensitive to the isomers of compounds. This study can provide a theoretical basis for studying the changes in the characteristics of sea buckthorn juice during fermentation, particularly with regard to its flavor.

## Figures and Tables

**Table 1:** The flavor compounds presented in the sea buckthorn juice during fermentation by GC–IMS.

Compound		CAS#		Formula		MW	RI	Rt [s]	Dt [a.u.]
Esters	Ethyl hexanoate (D)	C123660		C_8_H_16_O_2_		144.20	1002.00	570.77	1.81
	Ethyl 2-methylbutanoate	C7452791		C_7_H_14_O_2_		130.20	844.50	316.61	1.66
	Isopentyl isovalerate (D)	C659701		C_10_H_20_O_2_		172.30	1102.80	769.43	2.03
	Ethyl acetate	C141786		C_4_H_8_O_2_		88.10	581.90	138.26	1.34
	Ethyl butyrate	C105544		C_6_H_12_O_2_		116.20	788.30	259.77	1.56
	Isopentyl isovalerate (M)	C659701		C_10_H_20_O_2_		172.30	1107.40	780.01	1.45
	Ethyl hexanoate (M)	C123660		C_8_H_16_O_2_		144.20	1002.00	570.77	1.34
	Ethyl octanoate (M)	C106321		C_10_H_20_O_2_		172.30	1182.30	973.89	1.48
	Ethyl 2-methylpropanoate	C97621		C_6_H_12_O_2_		116.20	744.40	222.02	1.56
	Ethyl benzoate (M)	C93890		C_9_H_10_O_2_		150.20	1155.80	900.17	1.27
	Isopentyl hexanoate (M)	C2198610		C_11_H_22_O_2_		186.30	1226.90	1111.21	1.53
	Ethyl benzoate (D)	C93890		C_9_H_10_O_2_		150.20	1156.90	903.19	1.73
	Ethyl pentanoate (D)	C539822		C_7_H_14_O_2_		130.20	898.00	383.07	1.68
	Ethyl octanoate (D)	C106321		C_10_H_20_O_2_		172.30	1181.70	971.98	2.03
	(E)-2-Hexenyl acetate	C2497189		C_8_H_14_O_2_		142.20	1021.70	605.03	1.86
	Propyl acetate	C109604		C_5_H_10_O_2_		102.10	714.90	199.75	1.48
	Ethyl pentanoate (M)	C539822		C_7_H_14_O_2_		130.20	901.20	387.93	1.27
	Isopentyl butanoate (M)	C106274		C_9_H_18_O_2_		158.20	1065.00	687.81	1.41
	Ethyl propanoate	C105373		C_5_H_10_O_2_		102.10	699.80	189.20	1.46
	Isopentyl acetate	C123922		C_7_H_14_O_2_		130.20	873.40	350.65	1.75
	Ethyl heptanoate	C106309		C_9_H_18_O_2_		158.20	1097.50	757.34	1.41
	Isopentyl hexanoate (D)	C2198610		C_11_H_22_O_2_		186.30	1227.80	1114.23	2.13
	Isobutyl acetate	C110190		C_6_H_12_O_2_		116.20	759.60	234.45	1.62
	Isopentyl butanoate (D)	C106274		C_9_H_18_O_2_		158.20	1064.80	687.52	1.94
Alcohols	Ethanol	C64175		C_2_H_6_O		46.10	432.70	94.29	1.13
	Isopentyl alcohol (M)	C123513		C_5_H_12_O		88.10	731.60	212.05	1.50
	Isopentyl alcohol (D)	C123513		C_5_H_12_O		88.10	731.10	211.68	1.79
	2-Methyl-1-propanol	C78831		C_4_H_10_O		74.10	620.10	152.48	1.38
Terpenes	Camphene (M)	C79925		C_10_H_16_		136.20	941.00	452.78	1.20
	Camphene (P)	C79925		C_10_H_16_		136.20	940.10	451.15	1.65
	Beta-Ocimene (M)	C13877913		C_10_H_16_		136.20	1056.30	670.39	1.21
	Beta-Ocimene (P)	C13877913		C_10_H_16_		136.20	1055.60	669.04	1.25
Ketone	6-Methyl-5-hepten-2-one	C110930		C_8_H_14_O		126.20	989.90	547.37	1.18
**Compound**		**Relative Contents (%)**							**Odor Description**
		**Fermentation Period**							
		**0 h**	**12 h**	**24 h**	**36 h**	**48 h**	**60 h**	**72 h**	
Esters	Ethyl hexanoate (D)	23.17 ±0.19 ^a^	22.80 ± 0.15 ^b^	19.39 ± 0.19 ^e^	21.26 ± 0.17 ^d^	20.87 ± 0.08 ^c^	20.76 ± 0.08 ^ef^	20.67 ± 0.03 ^f^	apple peel, fruity *
	Ethyl 2-methylbutanoate	15.78 ± 0.14 ^a^	15.59 ± 0.07 ^b^	14.23 ± 0.11 ^c^	14.29 ± 0.06 ^d^	14.66 ± 0.03 ^c^	14.47 ± 0.01 ^d^	14.51 ± 0.02 ^d^	Apple *
	Isopentyl isovalerate (D)	6.98 ± 0.24 ^cd^	7.16 ± 0.08 ^d^	7.18 ± 0.11 ^b^	7.44 ± 0.07 ^c^	7.62 ± 0.09 ^a^	7.81 ± 0.05 ^ab^	7.89 ± 0.09 ^ab^	Sweet, fruity, green, ripe, apple, jammy, tropical ^#^
	Ethyl acetate	6.47 ± 0.03 ^d^	5.98 ± 0.05 ^e^	9.43 ± 0.06 ^a^	8.36 ± 0.07 ^b^	8.01 ± 0.04 ^b^	8.11 ± 0.02 ^c^	8.13 ± 0.08 ^c^	pineapple *
	Ethyl butyrate	5.73 ± 0.08 ^a^	5.72 ± 0.02 ^b^	4.35 ± 0.06 ^d^	4.40 ± 0.07 ^e^	4.66 ± 0.01 ^c^	4.26 ± 0.01 ^f^	4.30 ± 0.01 ^f^	apple *
	Isopentyl isovalerate (M)	5.06 ± 0.02 ^a^	5.13 ± 0.01 ^b^	3.79 ± 0.07 ^c^	3.90 ± 0.07 ^d^	3.73 ± 0.01 ^d^	3.97 ± 0.08 ^d^	3.80 ± 0.03 ^e^	Sweet, fruity, green, ripe, apple, jammy, tropical ^#^
	Ethyl hexanoate (M)	3.35 ± 0.15 ^a^	3.24 ± 0.02 ^b^	2.12 ± 0.04 ^c^	2.22 ± 0.02 ^cd^	2.05 ± 0.02 ^de^	2.07 ± 0.07 ^ef^	2.02 ± 0.01 ^f^	apple peel, fruity *
	Ethyl octanoate (M)	2.76 ± 0.11 ^c^	2.95 ± 0.03 ^bc^	2.84 ± 0.08 ^ab^	2.95 ± 0.04 ^bc^	2.98 ± 0.03 ^a^	3.10 ± 0.02 ^ab^	3.09 ± 0.00 ^ab^	winey, sweet, apricot, banana, brandy, pear ^#^
	Ethyl 2-methylpropanoate	2.70 ± 0.03 ^b^	2.86 ± 0.01 ^a^	2.09 ± 0.01 ^c^	1.89 ± 0.01 ^d^	2.17 ± 0.08 ^c^	1.90 ± 0.01 ^d^	1.92 ± 0.01 ^d^	rubber *
	Ethyl benzoate (M)	2.66 ± 0.05 ^a^	2.67 ± 0.02 ^b^	2.14 ± 0.03 ^c^	2.16 ± 0.05 ^d^	2.09 ± 0.03 ^d^	2.16 ± 0.01 ^d^	2.18 ± 0.02 ^d^	chamomile *
	Isopentyl hexanoate (M)	1.04 ± 0.04 ^a^	1.05 ± 0.00 ^a^	0.83 ± 0.03 ^b^	0.89 ± 0.01 ^b^	0.85 ± 0.02 ^b^	0.90 ± 0.01 ^b^	0.87 ± 0.02 ^b^	banana, apple, pineapple, green ^#^
	Ethyl benzoate (D)	1.01 ± 0.06 ^bc^	1.00 ± 0.02 ^c^	1.00 ± 0.05 ^b^	1.06 ± 0.02 ^bc^	1.13 ± 0.02 ^a^	1.18 ± 0.01 ^a^	1.19 ± 0.02 ^a^	camomile *
	Ethyl pentanoate (D)	0.98 ± 0.04 ^a^	0.86 ± 0.01 ^b^	0.66 ± 0.02 ^c^	0.65 ± 0.00 ^d^	0.82 ±0.01 ^b^	0.66 ± 0.01 ^d^	0.68 ± 0.01 ^cd^	yeast, fruit *
	Ethyl octanoate (D)	0.81 ± 0.05 ^d^	0.89 ± 0.01 ^d^	1.28 ± 0.04 ^c^	1.46 ± 0.03 ^b^	1.68 ± 0.07 ^a^	1.77 ± 0.02 ^a^	1.83 ± 0.04 ^a^	winey, sweet, apricot, banana, brandy, pear ^#^
	(E)-2-Hexenyl acetate	0.78 ± 0.01 ^ab^	0.75 ± 0.01 ^c^	0.75 ± 0.01 ^b^	0.77 ± 0.01 ^c^	0.80 ± 0.00 ^a^	0.83 ± 0.02 ^ab^	0.82 ± 0.02 ^ab^	Sweet, apple, banana ★
	Propyl acetate	0.39 ± 0.01 ^c^	0.21 ± 0.01 ^e^	0.26 ± 0.01 ^d^	0.54 ± 0.04 ^a^	0.46 ± 0.01 ^b^	0.50 ± 0.00 ^b^	0.54 ± 0.01 ^a^	pear ★
	Ethyl pentanoate (M)	0.36 ± 0.00 ^b^	0.39 ± 0.01 ^a^	0.12 ± 0.01 ^c^	0.14 ± 0.01 ^c^	0.13 ± 0.01 ^c^	0.13 ± 0.00 ^c^	0.13 ± 0.01 ^c^	yeast, fruit *
	Isopentyl butanoate (M)	0.30 ± 0.01 ^a^	0.29 ± 0.00 ^b^	0.22 ± 0.00 ^cd^	0.23 ± 0.01 ^d^	0.24 ± 0.01 ^c^	0.25 ± 0.01 ^c^	0.24 ± 0.01 ^cd^	green, apricot, pear, banana ^#^
	Ethyl propanoate	0.21 ± 0.01 ^e^	0.18 ± 0.01 ^f^	2.26 ± 0.01 ^c^	2.49 ± 0.02 ^b^	2.50 ± 0.01 ^a^	2.23 ± 0.01 ^d^	2.21 ± 0.02 ^d^	yeast, fruit *
	Isopentyl acetate	0.20 ± 0.00 ^f^	0.18 ± 0.01 ^f^	4.98 ± 0.03 ^a^	2.35 ± 0.02 ^b^	2.01 ± 0.00 ^c^	1.98 ± 0.01 ^d^	1.94 ± 0.01 ^e^	banana *
	Ethyl heptanoate	0.18 ± 0.00 ^d^	0.18 ± 0.00 ^d^	0.16 ± 0.00 ^e^	0.19 ± 0.01 ^c^	0.19 ± 0.00 ^bc^	0.20 ± 0.00 ^b^	0.21 ± 0.00 ^a^	pineapple, cognac, rummy, winey ^#^
	Isopentyl hexanoate (D)	0.11 ± 0.01 ^b^	0.13 ± 0.01 ^ab^	0.11 ± 0.00 ^b^	0.13 ± 0.02 ^ab^	0.14 ± 0.01 ^a^	0.13 ± 0.00 ^ab^	0.14 ± 0.01 ^ab^	banana, apple, pineapple, green ^#^
	Isobutyl acetate	0.05 ± 0.00 ^e^	0.06 ± 0.01 ^e^	1.45 ± 0.01 ^a^	0.34 ± 0.00 ^c^	0.34 ± 0.00 ^b^	0.29 ± 0.00 ^d^	0.29 ± 0.00 ^d^	fruit, pple, banana *
	Isopentyl butanoate (D)	0.04 ± 0.00 ^ab^	0.04 ± 0.00 ^ab^	0.04 ± 0.00 ^ab^	0.04 ± 0.00 ^b^	0.04 ± 0.00 ^a^	0.04 ± 0.00 ^ab^	0.04 ± 0.00 ^ab^	fruity, green, apricot, pear, banana ^#^
Alcohols	Ethanol	7.59 ± 0.02 ^e^	8.03 ± 0.02 ^d^	8.06 ± 0.04 ^a^	8.59 ± 0.03 ^ab^	8.31 ± 0.05 ^a^	8.64 ± 0.03 ^bc^	8.62 ± 0.01 ^c^	sweet *
	Isopentyl alcohol (M)	2.04 ± 0.03 ^bc^	2.20 ± 0.06 ^a^	1.82 ± 0.03 ^d^	2.10 ± 0.01 ^c^	2.02 ± 0.03 ^c^	2.19 ± 0.01 ^ab^	2.16 ± 0.02 ^bc^	whiskey, malt, burnt *
	Isopentyl alcohol (D)	0.23 ± 0.01 ^e^	0.29 ± 0.00 ^d^	0.49 ± 0.01 ^c^	0.57 ± 0.00 ^b^	0.54 ± 0.01 ^b^	0.59 ± 0.00 ^a^	0.60 ± 0.01 ^a^	
	2-Methyl-1-propanol	0.18 ± 0.01 ^d^	0.23 ± 0.02 ^c^	0.14 ± 0.01 ^e^	0.32 ± 0.00 ^ab^	0.30 ± 0.00 ^b^	0.34 ± 0.00 ^ab^	0.34 ± 0.01 ^a^	wine, solvent, bitter *
Terpenes	Camphene (M)	0.43 ± 0.01 ^a^	0.37 ± 0.01 ^b^	0.31 ± 0.02 ^c^	0.28 ± 0.00 ^e^	0.35 ± 0.00 ^b^	0.30 ± 0.00 ^de^	0.30 ± 0.00 ^d^	camphor *
	Camphene (P)	0.48 ± 0.01 ^a^	0.49 ± 0.02 ^a^	0.36 ± 0.00 ^b^	0.35 ± 0.00 ^c^	0.36 ± 0.00 ^b^	0.34 ± 0.01 ^c^	0.35 ± 0.00 ^c^	
	Beta-Ocimene (M)	0.36 ± 0.02 ^a^	0.37 ± 0.01 ^a^	0.31 ± 0.01 ^b^	0.31 ± 0.00 ^cd^	0.31 ± 0.01 ^bc^	0.30 ± 0.00 ^d^	0.31 ± 0.00 ^cd^	Citrus, tropical, green, woody ^#^
	Beta-Ocimene (P)	0.29 ± 0.01 ^ab^	0.30 ± 0.00 ^a^	0.26 ± 0.01 ^c^	0.28 ± 0.00 ^c^	0.27 ± 0.00 ^c^	0.29 ± 0.01 ^bc^	0.29 ± 0.00 ^c^	
Ketone	6-Methyl-5-hepten-2-one	0.25 ± 0.01 ^d^	0.28 ± 0.01 ^d^	0.44 ± 0.01 ^c^	0.47 ± 0.01 ^c^	0.51 ± 0.01 ^b^	0.54 ± 0.01 ^ab^	0.55 ± 0.00 ^a^	pepper, mushroom, rubber *

Note: Molecular weight (MW), reserved index (RI), retention time (Rt), drift time (Dt), monomer (M), dimer (D) and polymer (P). Different letters in the same row indicate statistically significant differences in the results (ρ < 0.05). * Odor descriptions from the website http://www.flavornet.org/flavornet.html (accessed on 1 September 2022). ^#^ Odor descriptions from reference [14]. ★ Odor descriptions from the website https://foodb.ca/compounds (accessed on 1 September 2022).

## Data Availability

The data that support the findings of this study are available from the corresponding author upon reasonable request.

## Supplementary Materials

The following supporting information can be downloaded at: https://www.mdpi.com/article/10.3390/foods11213471/s1, Table S1: Sensory evaluation criteria and intensity for sea buckthorn fermented juice.
