# Supplementary material for: Changes of Volatile Flavor Compounds in Sea Buckthorn Juice during Fermentation Based on Gas Chromatography–Ion Mobility Spectrometry
Source: Foods. 2022 Nov 1;11(21):3471. doi: 10.3390/foods11213471 (PMC9655934; doi:10.3390/foods11213471)
Supplement: Supplementary file 1 [file foods-11-03471-s001.zip › foods-1937232-supplementary (1).pdf]

Supplementary material

Table S1. Sensory evaluation criteria and intensity for sea buckthorn fermented juice

Sensory attributes	Criteria	Intensity
Fruity	Pure fruity of sea buckthorn	9
	Light fruity of sea buckthorn	4.5
	Not obvious aroma or peculiar smell	0
Appearance	Normal orange, no stratification	9
	Normal orange, stratified	4.5
	Orange become light, layered	0
Sweet and sour	Sour or sweet suitable, mild taste	9
	More sweet or more sour	4.5
	Hard to accept	0
Sweet odor	Smells pure honey sweet	9
	Smells slight honey sweet	4.5
	Not obvious	0
Bitterness	Not obvious	9
	Light	4.5
	Heavy	0
